# Which games are growing bacterial populations playing?

**DOI:** 10.1098/rsif.2015.0121

**Published:** 2015-07-06

**Authors:** Xiang-Yi Li, Cleo Pietschke, Sebastian Fraune, Philipp M. Altrock, Thomas C. G. Bosch, Arne Traulsen

**Affiliations:** 1 Department of Evolutionary Theory, Max Planck Institute for Evolutionary Biology, August-Thienemannstraße 2, 24306 Plön, Germany; 2 Zoological Institute, Christian-Albrechts-University Kiel, Olshausenstraße 40, 24098 Kiel, Germany; 3 Dana-Farber Cancer Institute, Boston, MA 02215, USA; 4 Harvard T.H. Chan School of Public Health, Boston, MA 02115, USA; 5 Program for Evolutionary Dynamics, Harvard University, Cambridge, MA 02138, USA

**Keywords:** bacterial interactions, frequency-dependent selection, Lotka–Volterra equations, nonlinear dynamics, population dynamics

## Abstract

Microbial communities display complex population dynamics, both in frequency and absolute density. Evolutionary game theory provides a natural approach to analyse and model this complexity by studying the detailed interactions among players, including competition and conflict, cooperation and coexistence. Classic evolutionary game theory models typically assume constant population size, which often does not hold for microbial populations. Here, we explicitly take into account population growth with frequency-dependent growth parameters, as observed in our experimental system. We study the *in vitro* population dynamics of the two commensal bacteria (*Curvibacter* sp. (AEP1.3) and *Duganella* sp. (C1.2)) that synergistically protect the metazoan host *Hydra vulgaris* (AEP) from fungal infection. The frequency-dependent, nonlinear growth rates observed in our experiments indicate that the interactions among bacteria in co-culture are beyond the simple case of direct competition or, equivalently, pairwise games. This is in agreement with the synergistic effect of anti-fungal activity observed *in vivo*. Our analysis provides new insight into the minimal degree of complexity needed to appropriately understand and predict coexistence or extinction events in this kind of microbial community dynamics. Our approach extends the understanding of microbial communities and points to novel experiments.

## 1. Introduction

From protists to humans, all animals and plants are inhabited by microbial organisms. There is an increasing appreciation that these resident microbes influence evolutionary important traits (e.g. fitness) of their hosts [1,2]. The host and its associated microbiota do not evolve independently, but form a metaorganism that evolves as a whole under natural selection [3,4]. The microbial organisms affect host fitness in various ways. They contribute by enhancing digestion and mediating energy balance via nutrient absorption [5–8], controlling pathogenic reactions [9–11], stimulating stem cell turnover [12] and the maturation of immune systems [13,14] and potentially triggering normal development of organs [15,16]. They even promote hybrid lethality/sterility and thus contribute to speciation [4]. Despite the well-accepted importance of microbiota to the normal functioning of the host, the molecular and cellular mechanisms controlling interactions within the metaorganisms are poorly understood and many key interactions between the associated organisms remain unknown [17]. Moreover, the applications of theoretical frameworks to improve the understanding of the ecological interactions among microbiota and between host and microbiota are still very limited. Notable exceptions include [18–22].

There exists an established body of ecological theory for the population dynamics of large organisms, such as plants and animals. But whether these theories also apply to the dynamics of microorganisms is largely unknown, yet seldom questioned [17]. Compared to large organisms, bacteria not only differ greatly in the small physical size and high growth rate, but also in the drastic changes of population size. These differences are most profound during neonatal host development, while the microbiota establish. There has been comparably little theoretical development regarding the interaction dynamics of size fluctuating bacterial populations [23,24]. A recent study showed that microbiota colonization patterns during host development display complex dynamics, which cannot be explained with standard models of density independent competition [25]. This study also showed that the microbiota composition in fresh water *Hydra* hatchlings changed greatly during development. A highly variable initial stage was followed by a transient adult-like phase in which the microbial composition was temporarily very similar to the stable adult microbiota, yet only retained for a short time. The adult microbiota composition only reappear after further drastic changes. Remarkably, a similar ‘in-out-in’ colonization pattern of adult-like microbiota composition was also observed in human infants [26].

Even more interestingly, the complex microbiota in *Hydra* prevents infection by the filamentous fungus *Fusarium* sp. [11]. Using a germ-free *Hydra* model, it was shown that germ-free polyps were highly susceptible to fungal infection, while restoring the complex microbiota in gnotobiotic polyps prevented infection. Testing single bacterial isolates from *Hydra* in mono-associations revealed that none of the tested bacterial colonizers alone was able to provide full anti-fungal resistance. By contrast, resistance, as observed in control polyps, was achieved in polyps di-associated with the two most dominant bacterial colonizers, *Curvibacter* sp. and *Duganella* sp., by exhibiting a strong synergistic effect [11]. This finding provides compelling evidence for the importance of bacteria–bacteria interactions in the normal functioning of *Hydra*-associated microbiota.

Besides empirical evidence, ecological theories are indispensable to our understanding of the full complexity in host–microbiota interplay. Evolutionary game theory takes its root in classic game theory but focuses on the frequency dynamics of strategies in populations instead of the quality of competing strategies themselves. Therefore, it is especially suited for studying microbial population dynamics on the ecological timescale, where the fitness landscapes of different types are constantly changing [27], and thus selection is frequency dependent [28–30]. In the case of deterministic dynamics, there exists a strong link between game dynamics and ecological dynamics. The game theoretical replicator equation is mathematically closely related to the ecological Lotka–Volterra equation with linear growth rates [31]. The replicator equation focuses on relative changes in population size under frequency-dependent fitness, which can provide conceptual insights into the microscopic interactions between individual bacteria cells. The Lotka–Volterra equation describes populations of changing size. Therefore, it can be conveniently linked to experiments [32–34]. In the case of two interacting species, replicator dynamics and the competitive Lotka–Volterra equations predict competitive exclusion, i.e. extinction of one type, or coexistence at a unique state of population composition.

Considering the classic Lotka–Volterra equation as the potential framework to understand bacterial interaction reveals limitations in modelling the population dynamics of the two most abundant bacterial species that interact synergistically to protect the *Hydra* host from pathogenic infection. We show that the patterns observed in our experiments require a more detailed consideration of bacteria–bacteria interactions. This includes growth rates that nonlinearly depend on the relative abundance of different species in the exponential growth phase.

Compared to human and other more complex model species, the early metazoan *Hydra* provides a comparably simple system with a genetically determined bacterial community colonizing the surface of the ectodermal epithelium [35]. Therefore, it serves as a powerful model organism for studying the interactions between the host and its bacterial community [36,37]. In addition, the synergistic interaction between the two dominant bacteria species is especially exciting. It provides anti-fungal protection to the host that cannot be achieved by either of the bacteria when associated with the host alone [11]. In a first attempt to quantitatively understand the host–microbial interaction, here we determine the mechanisms of bacterial interactions without the host's influence. We performed double culture experiments *in vitro* with the two most abundant actors in the *Hydra* microbiota. In order to develop a mathematical model for this scenario, here we propose general principles governing the interactions within the microbiota. We start by adding frequency-dependent growth rates into the Lotka–Volterra framework and infer the possible dynamics for linear and quadratic frequency-dependent growth rates, and then put these results into the context of our empirical data. These data suggest interactions between multiple players as one possible mechanism of the interactions among individual bacterial cells, which can lead to global population dynamics qualitatively similar to those observed in our experiments.

## 2. Material and methods

We study the interactions between the two species of Betaproteobacteria *Curvibacter* sp. AEP1.3 (*C*) and *Duganella* sp. C1.2 (*D*). Both bacteria belong to the order of Burkholderiales, while *C* represents a Comamonadaceae and *D* an Oxalobacteraceae [11]. We chose these two bacteria because (i) they are naturally found in the bacterial community of the freshwater polyp *Hydra vulgaris* (AEP) and are the two most abundant species in the microbiota (*C*: 75.6% and *D*: 11.1%) [11]; (ii) the synergistic interaction between the two bacteria species effectively provides anti-fungal protection for the host [11]; and (iii) the morphology of their colonies can be distinguished from each other on agar plates.

### 2.1. Monoculture and double culture experiments

**Figure 1.**
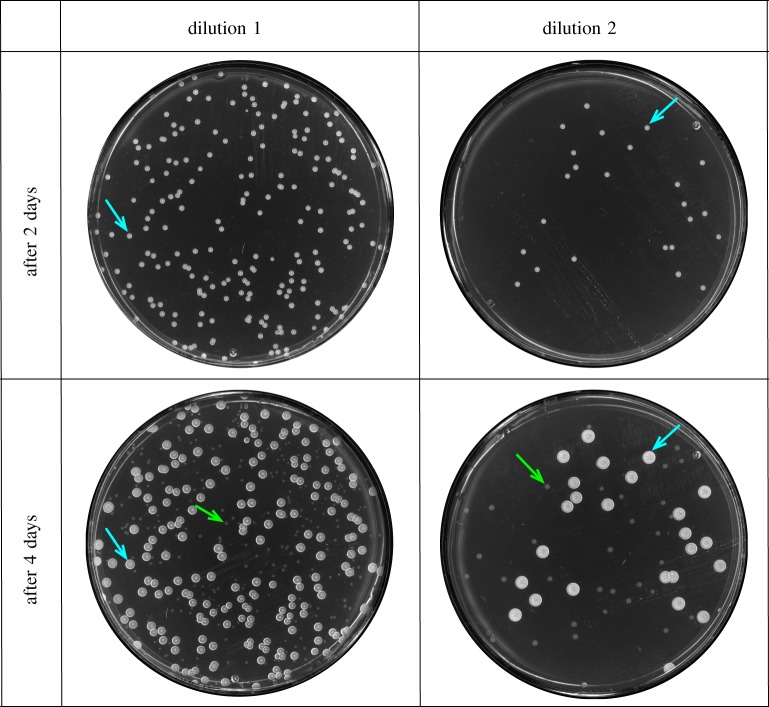
We quantify the density of *Curvibacter* sp. and *Duganella* sp. by the method of counting colony forming units (cfu). At each time point, we took a sample of the bacterial culture and performed a series of dilutions. The diluted samples were then plated on a Petri dish with solid agar medium. After 2 days, the fast-growing *Duganella* sp. (indicated by cyan arrows) already formed clear and distinct colonies on the plates. After 4 days, the slow-growing *Curvibacter* sp. (indicated by green arrows) also formed distinct colonies. Under the assumption that one colony was formed by one single founder cell in the medium, we can calculate the cell densities of each of the two bacteria species in the original sample by adjusting the numbers of colonies with corresponding dilution factors.

To determine the growth rate in monocultures, we inoculated for each bacterium 50 ml R2A medium with 10 concentrations between 2.0 × 10^3^ and 1.0 × 10^5^ cfu ml^–1^ of *C* or *D* of an overnight culture. The cell numbers of each bacteria were estimated by counting the colony forming units (cfu) and were cross-checked with optical density (OD) measurements at OD600 = 0.1 (1.0 × 10^8^ cfu ml^–1^ for *C* and 2.0 × 10^7^ cfu ml^–1^ for *D*). In double culture experiments, we kept a total initial concentration of 1.0 × 10^5^ cfu ml^–1^ and applied a gradient of different initial frequencies of species *C* and *D*. Over the course of 3 days, three times a day, we measured the OD600 of the cultures and plated two dilutions, which were adjusted individually to the OD, on R2A agar plates. After 2 days, we counted the number of colonies of *D* and after 4 days those of *C*. This difference in counting times was due to different growth rates of the two bacteria on agar plates (figure 1).

### 2.2. Data preparation and analysis

For cell culture growth over time in both monocultures and double cultures, we calculated growth rate functions by applying a linear regression to the log-linear transformed data. The goodness of fit was calculated using the (adjusted) *R*^2^ [38,39]. In monoculture experiments for both species, we tested the dependency of growth rate in the exponential growth phase on a gradient of initial cell densities.

In double culture experiments for both species, we tested linear, quadratic and cubic functions of growth rate on a gradient of initial frequencies. These three different model hypotheses were then compared using Akaike information criterion (AIC) [40] and Bayesian information criterion (BIC) [41,42], based on the likelihood functions of a normally distributed error term in the linear regression model. The most appropriate models were chosen with the agreement between both AIC and BIC tests.

In double culture experiments, if the cell density value was missing for one of the species at a certain point in time during the exponential growth phase, this data point was excluded when plotting the growth trajectories. But the cell density of the species that did have a valid count can still be used for calculating the growth rate.

## 3. Mathematical model

**Figure 2.**
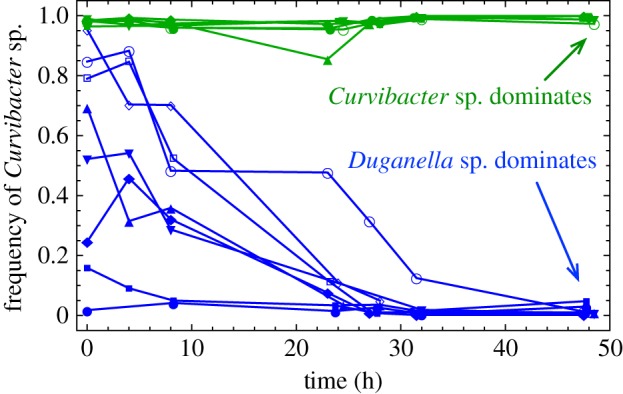
Change in the frequency of *Curvibacter* sp. in double cultures with *Duganella* sp. The frequency of *Curvibacter* sp. approaches the 0 or 1 boundaries over time, depending on the initial frequency of both types. If the culture was inoculated with a high frequency of *Curvibacter* sp., the frequency of it remains high (green trajectories), otherwise *Duganella* sp. quickly outgrows and eventually pushes the frequency of *Curvibacter* sp. towards 0 (blue trajectories). This resembles the dynamics in a coordination game, where the two homogeneous populations are stable.

Our mathematical model is directly motivated by experimental observations. We analysed the growth trajectories of the double culture experiments of *Curvibacter* sp. (*C*) and *Duganella* sp. (*D*), the two most abundant species that interact synergistically to protect the *Hydra* host from fungal infection. Depending on the initial condition, one of the two species eventually becomes dominant in frequency (figure 2). This resembles a coordination game [43], which is characterized by an unstable intermediate fixed point and two stable boundary fixed points. However, the submissive species does not go extinct but keeps growing in absolute density. This is a key feature, which is usually neglected in game theoretic models where only changes in frequency, fixation and respective extinction are considered.

Based on these experimental findings, we propose a mathematical model building on the classic Lotka–Volterra competition dynamics, which is mathematically closely related to the replicator equation in game theory models [31]. In our model, the maximum growth rates in the exponential growth phase of the bacterial culture are frequency dependent. This model of *in vitro* bacterial interactions in growing populations serves as a basis for making comparisons with the *in vivo* scenarios influenced by the host.

### 3.1. Lotka–Volterra competition model with linear frequency-dependent growth rates

The population dynamics in two-species Lotka–Volterra competition models has been thoroughly discussed in textbooks [32,33]. Those models assume that the two species only compete for the same limited resources, e.g. the same food, or space with unrestricted nutrient provision, or territory which is directly related to food resources, but the two species do not interact otherwise. Then the dynamics are given by the following growth equations:3.1
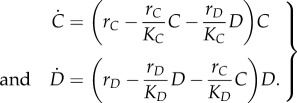


Both species exhibit logistic growth when cultured alone. The value of growth rates *r* and carrying capacities *K* are positive constants. Equations (3.1) predict that the two species can coexist only when exactly *r_C_K_C_* = *r_D_K_D_*. Otherwise, the species with higher *rK* value wins the competition, and the other species goes extinct (competitive exclusion).

We depart from this simple case and consider the case where growth rates *r_C_* and *r_D_* are frequency-dependent linear functions. The frequency of *C* is denoted as *x* (*x* = *C*/(*C* + *D*)), and the frequency of *D* is thus 1 − *x*, in the double culture system. Therefore, *r_C_* and *r_D_* can be written as linear functions of *x*3.2
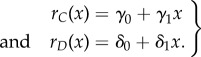


Since the growth rates *r_C_* and *r_D_* are the maximum growth rates reached at the exponential growth phase, they are always positive. Consequently, we require *γ*_0_, *δ*_0_, *γ*_0_ + *γ*_1_, and *δ*_0_ + *δ*_1_ to be positive. Then the population dynamics is described by a set of modified Lotka–Volterra equations, with linear frequency-dependent growth rates and interaction coefficients. This is equivalent to logistic growth functions with frequency-dependent competition coefficients. In the following, we assume constant carrying capacity for both species, *K_C_* = *K_D_* = *K*. For constant and linear growth rates, it is straightforward to generalize our findings to *K*_*C*_ ≠ *K*_*D*_. There are four steady states—three boundary cases (0, 0), (0, *K*), (*K*, 0), and the mixed solution:3.3
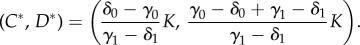


**Figure 3.**
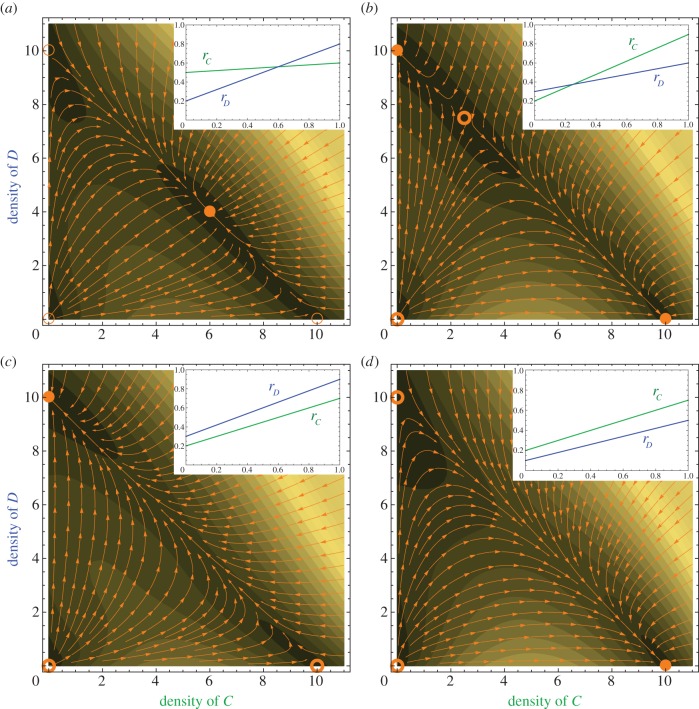
Population growth with linear frequency-dependent growth rates, cf. equations (3.2). The four main panels show the population dynamics. The insets show the corresponding linear frequency-dependent growth rate functions, in which the *x*-axis represents the frequency of *C*. In the main panels, filled circles are stable fixed points and open circles are unstable fixed points. The orange arrows show the stream line trajectories of the population dynamics starting from a variety of initial states. The gradient background captures the speed of change. (*a,b*) The growth rate functions cross in the range (0,1), leading to a mixed steady state (C^*^,D^*^). (*a*) If *r_C_*(0) > *r_D_*(0) and *r_C_*(1) < *r_D_*(1), (C^*^,D^*^) is stable (*r_C_* = 0.5 + 0.1*x*, *r_D_* = 0.2 + 0.6*x*)). (*b*) If *r_C_*(0) < *r_D_*(0) and *r_C_*(1) > *r_D_*(1), (C^*^,D^*^) is unstable (*r_C_* = 0.2 + 0.7*x*, *r_D_* = 0.3 + 0.3*x*). (*c*) If *r_C_*(*x*) < *r_D_*(*x*) for all *x*, there is no interior fixed point and only (0, *K*) is stable (*r_C_* = 0.2 + 0.5*x*, *r_D_* = 0.3 + 0.6*x*). (*d*) If *r_C_*(*x*) > *r_D_*(*x*) for all *x*, there is no interior fixed point and only (*K*, 0) is stable (*r_C_* = 0.2 + 0.5*x*, *r_D_* = 0.1 + 0.4*x*).

The mixed solution is only biologically meaningful if 0 < C^*^, D^*^ < 1. The relation C^*^ > 0 implies that the growth rates of the two species have to intersect at an intermediate frequency. For the overall dynamics, there are four qualitatively different cases, as illustrated in figure 3.

The set of points *C* + *D* = *K* satisfy the condition *Ċ* + *Ḋ* = 0; therefore, it is an invariant manifold [31,44] of the dynamics. Once this invariant manifold has been reached, the population dynamics will not lead away from it. Let *f* = |*C*_0_ + *D*_0_ –*K*| be the distance from any point (*C*_0_, *D*_0_) on the plane spanned by *C* and *D* to this invariant manifold. This distance decreases monotonically regardless of the initial condition (*C*_0_, *D*_0_) off the *C* + *D* = *K* manifold3.4
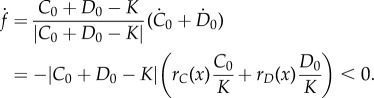


Note that this holds regardless of the functional form of the original frequency-dependent growth rates used in our model *r_C_*(*x*) and *r_D_*(*x*).

We can determine the local stability of the fixed points from the signs of the eigenvalues of the Jacobian matrix at the fixed points [31,45]. The fixed point at (0, *K*) is stable if *γ*_0_ < *δ*_0_. The fixed point at (*K*, 0) is stable if *γ*_0_ + *γ*_1_ > *δ*_0_ + *δ*_1_. Intuitively, these points are stable if the resident type grows faster than the invading type. In figure 3*a*, we illustrate the case where the growth rates *r_C_*(*x*) and *r_D_*(*x*) intersect in the interval (0, 1), and *r_C_*(0) > *r_D_*(0). Then the fixed points where one species goes extinct (*K*, 0) and (0, *K*) are unstable. On the one-dimensional stable manifold *C* + *D* = *K*, the stability of fixed points alternate. Hence, the internal fixed point (C^*^, D^*^) is stable. The other cases in figure 3*b*–*d* can be analysed in the same way.

### 3.2. Quadratic growth rates

In order to obtain multiple intersections of the frequency-dependent growth rates in the range (0, 1), the simplest possibility is that one of the growth rate functions is linear and the other is quadratic in species frequency. Therefore, we assume a quadratic term in the growth rate function of species *D*, and keep *r_C_*(*x*) and the population dynamics in equations (3.1) unchanged3.5
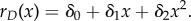


Depending on whether *r_D_*(*x*) has a maximum or a minimum, there are two different cases allowing the growth rate functions to intersect twice in the frequency range (0, 1). To have two intersections of *r_C_*(*x*) and *r_D_*(*x*) within (0,1), the function *r_C_*(*x*)−*r_D_*(*x*) must have two roots in the same range. Besides the two boundary solutions (0, *K*) and (*K*, 0), there are now two intermediate solutions on the manifold *C* + *D* = *K*, given by3.6
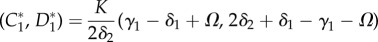
and3.7
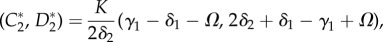
where 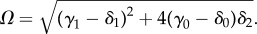


**Figure 4.**
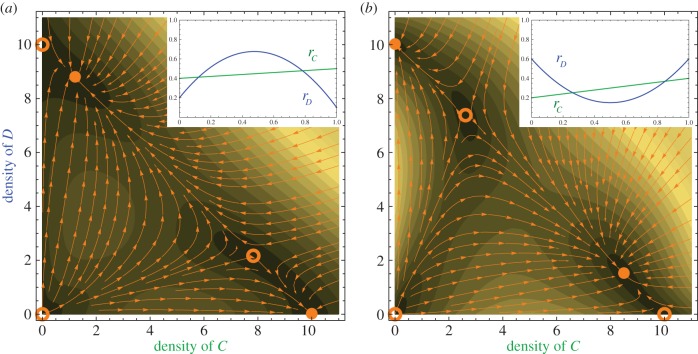
Population growth with quadratic growth functions (cf. equation (3.5)). Schematic phase trajectories near the steady states when frequency-dependent growth rate functions intersect twice. There is always a stable node (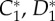) and a saddle point (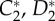) in the first quadrant. The insets represent the frequency-dependent growth rates, in which the *x*-axis is the frequency of *C*. There are two general cases in which *r_C_* and *r_D_* intersect twice. (*a*) The quadratic growth rate has a maximum due to *δ*_2_ < 0 (*r_C_* = 0.4 + 0.1*x*, *r_D_* = 0.2 + 2*x* − 2.1*x*^2^). (*b*) The quadratic growth rate has a minimum due to *δ*_2_ > 0 (*r_C_* = 0.2 + 0.2*x*, *r_D_* = 0.6 − 1.8*x* + 1.8*x*^2^).

Similar to the cases where *r_C_*(*x*) and *r_D_*(*x*) are linear functions of *x*, all trajectories lead to the invariant manifold *C* + *D* = *K*. The stability of fixed points alternates on this one-dimensional line. Conditioned on whether *r_D_*(*x*) has a maximum or a minimum, there are two different stability patterns on the manifold. The stability patterns are illustrated in figure 4. When the quadratic function *r_D_*(*x*) has a maximum (*δ*_2_ < 0), the two eigenvalues of the Jacobian matrix at (0, *K*) are −*δ*_0_ < 0 and *γ*_0_ – *δ*_0_ > 0. The two eigenvalues at (*K*, 0) are –*γ*_0_ – *γ*_1_ < 0 and –*γ*_0_ – *γ*_1_ + *δ*_0_ + *δ*_1_ + *δ*_2_ < 0. Therefore, (0, *K*) is unstable and (*K*, 0) is stable. Thus, the intermediate fixed point 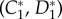 is stable and 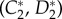 is unstable, as shown in figure 4*a*. The stability of fixed points when *r_D_*(*x*) has a minimum can be analysed in the same way, as shown in figure 4*b*. When the carrying capacity values *K_C_* and *K_D_* are not equal, the fixed points can still be calculated analytically, but 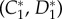 and 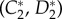 do not always both fall in the first quadrant.

## 4. Results

Let us now come to a comparison of our theoretical model and our experiments. The two bacteria species *Curvibacter* sp. and *Duganella* sp. were mixed with different initial frequency combinations and inoculated from a total concentration of 1.0 × 10^5^ cfu ml^–1^. Depending on the initial condition, the frequency of one of the species becomes much higher than the frequency of the other (figure 2), resembling the scenario of positive frequency-dependent selection in a coordination game. However, the absolute concentrations of both species increased over time in any case. The observed bacterial population dynamics are different from competitive exclusion in the sense that the advantageous species cannot entirely take over the population. The submissive species kept growing in absolute number despite the decrease in frequency.

**Figure 5.**
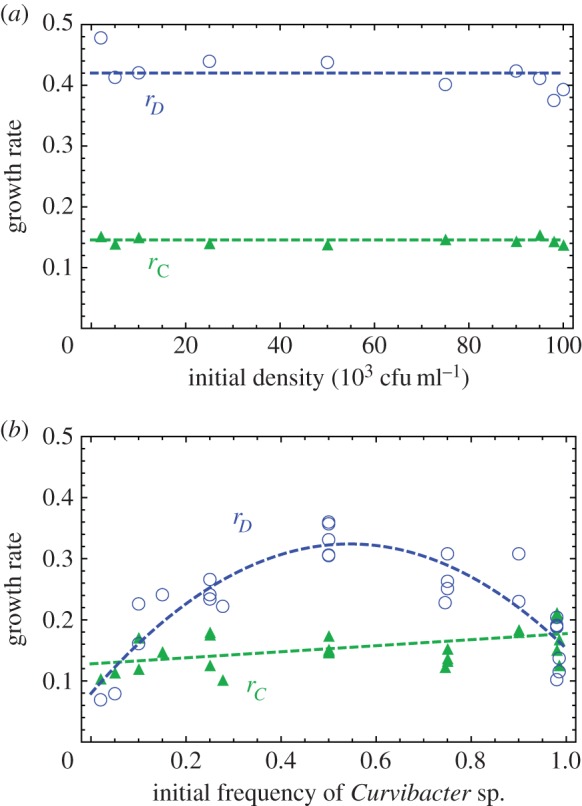
(*a*) The growth rates of *Curvibacter* sp. and *Duganella* sp. do not dependent on the initial density in monoculture experiments. *r_C_* = 0.146 (*R*^2^ = 0.998), *r_D_* = 0.420 (*R*^2^ = 0.996). (*b*) The growth rate during the exponential growth phase changes with initial frequencies in double cultures. This depends on the frequencies of both types, not necessarily in a linear fashion. The frequency-dependent growth rates of *Curvibacter* sp. were fitted to a linear function *r_C_* = 0.049*x* + 0.128 (adjusted *R*^2^ = 0.970), and those of *Duganella* sp. were fitted to a quadratic function *r_D_* = −0.825*x*^2^ + 0.898*x* + 0.080 (adjusted *R*^2^ = 0.972). The presence of *Duganella* sp. affects the growth rate of *Curvibacter* sp., but not substantially. By contrast, *Curvibacter* sp. has strong inhibiting effects on the growth rate of *Duganella* sp., across a wide range, even at very low frequency.

We first confirmed experimentally that the maximum growth rates of the two bacteria did not change with the initial density in monoculture experiments (figure 5*a*). The maximum growth rates of *Curvibacter* sp. and *Duganella* sp. in monoculture inoculated with a gradient of initial densities are fitted to constant functions *r_C_* = 0.146 (*R*^2^ = 0.998) and *r_D_* = 0.420 (*R*^2^ = 0.996). This suggests that differences in the growth rate in double cultures (figure 5*b*) depend on the frequency combinations of the two species, rather than their absolute densities.

**Figure 6.**
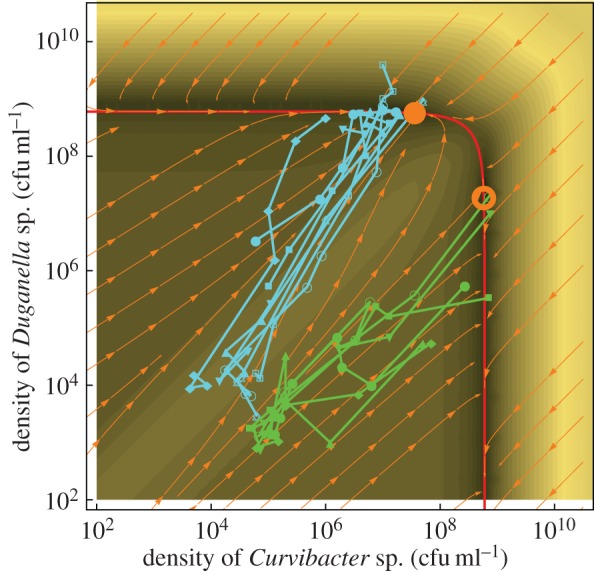
Growth trajectories plotted on the phase plane predicted by the model. The green trajectories lead to the unstable interior fixed point where *Curvibacter* sp. dominates in frequency. The cyan trajectories lead to the stable interior fixed point where *Duganella* sp. dominates. Filled and empty circles are stable and unstable fixed points, respectively. The red line is the invariant manifold *C* + *D* = *K*. The darkness of the contour plot represents the speed of change. The fast region along the diagonal represented by light colours separates the trajectories and pushes them to the two distinct fixed points in the interior. Near the fixed points the speed of change is very low; therefore, the two bacteria can coexist for a long time even near the internal unstable fixed point.

The frequency-dependent growth rate of *Curvibacter* sp. in double culture is fitted to a linear function *r_C_* = 0.049*x* + 0.128 (adjusted *R*^2^ = 0.970), and the growth rate of *Duganella* sp. in double culture is fitted to a quadratic function *r_D_* = −0.825*x* + 0.898*x* + 0.080 (adjusted *R*^2^ = 0.972), shown in figure 5*b*. For both *r_C_* and *r_D_*, we fitted the data to linear, quadratic and cubic functions. The models of best fit are chosen by both finite sample corrected AIC [40] and BIC [41,42]. The carrying capacity values *K* of both bacteria are 5.9 × 10^8^ cfu ml^–1^, which is the median of all double culture experiments (shown as the red line in figure 6).

From the comparison between growth rates in monoculture and double culture experiments, we observe that the presence of *Duganella* sp. does not profoundly influence the growth rates of *Curvibacter* sp., across a wide range of initial frequencies. But interestingly, the presence of *Curvibacter* sp. greatly influences the growth rate of *Duganella* sp., even at very low frequency (2%). When the initial frequency of *Curvibacter* sp. approaches 0 in double culture experiments, the growth rates of *Duganella* sp. do not converge to its growth rate when cultured alone. This disproportionally large effect of *Curvibacter* sp. on the system indicates its role as a keystone species [46,47] in the simplified microbiome.

Inserting the parameters measured from experiments, the phase plane dynamics obtained from equations (3.1) is shown in figure 6.

## 5. Discussion

Growth is important for bacterial populations. In comparison to larger organisms, the size of bacterial populations can fluctuate greatly and rapidly. Drastic changes occur particularly during the colonization of new environments. Such new environments can be abiotic systems like deep-sea hydrothermal vents [48,49], and biotic systems, including neonatal invertebrate hosts [25] and vertebrate hosts [26]. The composition of host-associated microbiota play an important role in the development and functioning of the host [50]. Prior work has shown that the colonization processes display fast growth and complex dynamics of the microbiota composition. Interesting examples include the in-out-in colonization pattern of the adult-like microbiota composition, observed in *Hydra* and human babies [26]. In addition, the ‘division of labour’-like dynamics in marine bacteria that colonize the chitin substratum [51], the life cycle-like dynamics of *Pseudomonas* biofilms that connect extrinsic structures and intrinsic protein expression [52], and cross-feeding and spatial partitioning of biofilm spaces between ecotypes [53] are also interesting examples of complex bacterial population dynamics during colonization of new environments. More examples of bacterial interactions in biofilms during the colonization of new environments can be found in the review by Hall-Stoodley *et al.* [54]. Despite the wealth of intriguing empirical discoveries, theoretical work that informs about principal mechanisms and leads to synthesis and integration of the available data is still limited [17].

Although various models have been developed to describe the time dynamics of bacterial growth, most of them only focus on the growth of one single species [55–57]. On the other hand, bacterial inter-species interactions are often studied in systems that have been intentionally excluded from notable fluctuations in population density [19,28,29]. In this way, species frequencies instead of absolute densities are used to describe population dynamics. For example, in the work of Kerr *et al.* [29], the authors studied the spatial interactions of three strains of *Escherichia coli*. It was found that when interactions are localized, the dynamics of the three strains resemble a ‘rock–paper–scissors’ game, with the colicin-resistant strain beating the colicin-producing strain, the colicin-producing strain beating the colicin-sensitive strain, and the colicin-sensitive strain outgrowing the colicin-resistant strain. This provided compelling support for game theoretical predictions in microbial ecology and evolution. However, the total population size was kept relatively constant on static agar plates. Therefore, frequency alone was sufficient to describe the population dynamics observed.

In contrast to populations that have been kept in constant size for theoretical convenience, here we analyse the dynamics in growing bacterial populations. We show that it is necessary to take into account both frequency and density to fully characterize the interaction dynamics. Considering microbial density alone, the growth curves of both *Curvibacter* sp. and *Duganella* sp. resemble logistic growth. Solely from the frequency perspective, the two bacteria seem to play a coordination game. Only in the interplay of the two effects, can we aptly describe the full picture. Our analysis reveals a system with two intermediate fixed points where both species coexist only if we look at both frequency and density dynamics together.

In evolutionary game theory, the vast majority of studies consider cases where the population size is either constant or where the system can be recast into a form where population growth does not affect the outcome. When it comes to growing populations—as in our case—many concepts and definitions become more cumbersome. Consequently, experimental and quantitative descriptions also become more delicate. For example, the typical definition of cooperative traits is that they increase the fitness of others at a fitness cost to self. But this increase could refer to the relative abundance or the absolute abundance. The former is commonly assumed in the evolutionary game theory literature, while the latter is commonly found in the experimental evolution literature. In other words, interactions could either influence the relative abundance or the absolute abundance in a variety of ways, which has not yet been fully addressed by theoretical models.

In classic logistic growth under Lotka–Volterra dynamics, or equivalently, game theoretical replicator dynamics, the growth rates in the exponential growth phase (simply denoted as growth rates in the following) are usually assumed to be constant [58–61]. The overall density-dependent change in population size can be introduced by altering the carrying capacity of the system. This alteration can arise from interspecific and intraspecific competition. For example, the carrying capacity could be determined by an evolutionary game [62]. In our case, we have seen that the growth rates are affected much more by bacterial interactions than the carrying capacities. Such growth rates can be frequency dependent due to interactions in the population other than direct competition [63].

A recent modelling study showed that social interactions between antibiotic-tolerant and antibiotic-sensitive bacteria can lead to patterns of multiple stability and thus explained how the microbiota composition can switch after antibiotic treatment [64]. Linear frequency-dependent growth rates have also been reported in a study of microbial random phase variation, which influences bacterial survival in changing environments [65]. A particularly interesting finding was reported by Trosvik *et al.* [66]. There the authors found a parabolic interaction relationship between *Bacteroides uniformis* and *Escherichia coli*, similar to our case. In our experimental system, the growth rate of *Duganella* sp. depends on the frequency of *Curvibacter* sp. also in a parabolic way. In the study by Trosvik *et al.*, *E. coli* had a positive effect on *B. uniformis* only to a certain point in the range of frequency, and then the effect turned out to be detrimental. Mechanistically, the positive effect of *E. coli* at low frequency might result from the removal of residual oxygen from the growth medium [67], given that *B. uniformis* is strictly anaerobic. But when the frequency of *E. coli* became higher, the authors argued that direct competition might outweigh the benefits from oxygen removal, and thus limited the growth of *B. uniformis* [66].

In our case, *Curvibacter* sp. has an overall suppression effect on the growth of *Duganella* sp. in double cultures. This effect is nonlinearly frequency dependent, most remarkably when the frequency difference of the two species is large. This is consistent with the situation *in vivo*. Inside the *Hydra* host, *Curvibacter* sp. has absolute dominance in abundance, despite *Duganella* sp.'s faster growth rate when cultured alone [11]. We have shown in the analysis that direct pairwise interactions between two bacterial cells are not enough to capture the complexity of the system. This leads us to consider the possibility that bacterial cells are involved in ‘multiplayer’ interactions [68–70]. In these scenarios, multiple bacterial cells interact with each other at the same time. Others have shown that bacteria can form nanotubes with multiple other cells and exchange molecules with each other [71,72]. This could be the physical basis of bacterial multiplayer games. In the simple system we have studied, at least a three-player game of degree two [73] is needed to account for the observed quadratic nonlinearity in frequency-dependent payoffs. Other alternative explanations include the effect of phage infection in the system. Our hypothesis is that the phage that infects *Curvibacter* sp. at an population equilibrium level may switch its host to *Duganella* sp. and thus induces an outbreak event that reduces its Malthusian growth rate by increased mortality. Testing this hypothesis is an extension of this project. Since frequency-dependent interactions can lead to multiple coexistence states [69], this effect could contribute to the tremendous microbial diversity coexisting with the numerically dominant few in natural environments, known as the ‘rare biosphere’ [74].

To conclude, in this study we show that it is necessary to consider both frequency and density dynamics in bacterial populations with noteworthy density fluctuations. Using a mathematical model, we examined the effects of linear and quadratic frequency-dependent growth rates in Lotka–Volterra dynamics. The interaction patterns of the system are richer with additional intermediate fixed points when frequency-dependent growth rate functions intersect more than once. Most importantly, our empirical data provide compelling evidence that the maximum growth rate can be a nonlinear function of frequency, without the effects of density limitation. This strongly indicates that there must be mechanisms other than direct pairwise interactions (including competition for a limiting resource) among the growing bacterial populations. Such complex patterns provide a mechanism for the maintenance of vast microbial diversity in the natural environment. Our study focuses on the interactions among growing bacterial populations without the effect of host or other environmental factors, serving as a null model for studying higher-level interactions.
